# Assessing the impact on caregivers of patients with schizophrenia: psychometric validation of the Schizophrenia Caregiver Questionnaire (SCQ)

**DOI:** 10.1186/s12888-016-0951-1

**Published:** 2016-07-18

**Authors:** Diana Rofail, Antoine Regnault, Stéphanie le Scouiller, Jérémy Lambert, Steven H. Zarit

**Affiliations:** Roche Products LTD, Shire Park, Welwyn Garden City, Hertfordshire UK; Patient-centered Outcomes, Mapi, 27 rue de la Villette, 69003 Lyon, France; Human Development and Family Studies, The Pennsylvania State University, University Park, PA USA

**Keywords:** Quality of life, Schizophrenia, Caregiver, Impact, Validation, Questionnaires

## Abstract

**Background:**

The Schizophrenia Caregiver Questionnaire (SCQ) was developed to assess the impact on caregivers of caring for patients with schizophrenia. The objective of this study was to develop a scoring algorithm for the SCQ, and evaluate its measurement properties.

**Methods:**

The SCQ was administered to 358 caregivers of patients with schizophrenia included in the observational PATTERN study of stabilized patients with persistent symptoms of schizophrenia receiving outpatient care. SCQ item selection and creation of scores were based on exploration of item response distribution, factor analyses, and Rasch model. Construct validity, reliability, and ability to detect change of the SCQ scores were investigated.

**Results:**

The final questionnaire comprised a ‘Humanistic impact’ supra-domain composed of a global score and four subdomain scores (‘Physical’; ‘Emotional’; ‘Social’; ‘Daily life’), and eight other domain scores related to the caregiving role (‘Exhaustion with caregiving’; ‘Feeling alone’; ‘Patient Dependence’; ‘Worries for the patient’; ‘Perception of caregiving’; ‘Financial dependence of the patient’; ‘Financial impact of caregiving’; ‘Overall difficulty of caregiving’). Two items from the SCQ were deleted. SCQ scores showed very good construct validity: Item convergent/discriminant validity were satisfactory; SCQ scores of caregivers of patients with more severe symptoms were higher indicating more impact (*p* < 0.05 for all scores); SCQ scores were meaningfully associated with measures of schizophrenia severity (PANSS and PSP) and caregivers’ Health-Related Quality of Life (Medical Outcome Survey Short Form 36 items). The SCQ Humanistic impact supra-domain scores demonstrated very good internal consistency reliability (Cronbach’s alphas between 0.80 and 0.96) and test-retest reliability (Intraclass Coefficient correlations ranging from 0.75 and 0.87); Other SCQ domain scores showed lower but still acceptable reliability coefficients. SCQ scores clearly increased for caregivers of patients whose schizophrenia worsened.

**Conclusions:**

Overall, the 30-item SCQ demonstrated very good measurement properties supporting its relevance to comprehensively measure the experience of caregivers of patients with schizophrenia.

## Background

Schizophrenia is a severe mental illness that affects between 0.3 and 0.7 % of the adult population worldwide and is considered a leading cause of disability [1]. Over the years, there has been a shift of care from psychiatric hospitals to outpatient treatment, community services, and informal caregivers. It is estimated that 50 to 90 % of people with a chronic psychiatric illness live with their families or friends [2, 3]. Caregivers, particularly informal caregivers, are defined as persons who have significant responsibility for managing the well-being of a person diagnosed with schizophrenia in an unpaid capacity. Caregivers provide an important service by reducing the need for formal care and the burden upon healthcare systems [4].

Even if caring for a person with schizophrenia may be considered a fulfilling and positive experience for some [5], it is also frequently associated with a negative impact on multiple aspects of a caregiver’s life. The impact of caregiving for a person with schizophrenia is a multidimensional concept, consisting of social, physiological, behavioral, functional, mental, medical and economic domains [6, 7]. Behavioral family management, psychoeducational family intervention, and family therapy have been shown to improve caregiver coping skills and reduce the impact of caregiving [8]. For example, Magliano et al. demonstrated that a psychoeducational family intervention contributed to improvements in caregivers’ experiences, especially in regards to coping with schizophrenia-related stigma [9].

The Zarit Burden Interview (ZBI) is a 22-item instrument which aims to assess the impact level experienced by a caregiver for an individual with dementia or disabilities [10]. The ZBI has been used in several studies investigating the impact of caregiving on caregivers for individuals with diverse conditions, including schizophrenia [11, 12]. However, as a generic measure, it is not specific to schizophrenia. Therefore the Schizophrenia Caregiver Questionnaire (SCQ) was recently developed as an adaptation of the ZBI to provide a comprehensive assessment of the impact of caregiving for an individual with schizophrenia [13]. The SCQ development process included a literature review and face to face open-ended, semi-structured, qualitative interviews with 19 US-English speaking caregivers of patients with schizophrenia [7, 13]. The concept elicitation part of the interviews showed that caring for a person with schizophrenia placed a significant impact on emotional, physical and financial lives of caregivers, as well as on their daily activities and relationships. The cognitive debriefing part confirmed the appropriateness and understanding by caregivers of the pilot version of the SCQ. Notable differences between the SCQ and ZBI included the specification of a recall period of ‘during the past 4 weeks’, the utilization of an 11-point numerical rating scale for all items, and additional questionnaire items. Moreover, the wording of some items was adapted to ensure that the questionnaire addressed issues of importance to caregivers of patients with schizophrenia. In addition, the cultural aspects of the measurement of impact on caregiver by the SCQ were explored, in the context of a linguistic validation of the questionnaire in 11 European languages to allow for the reliable pooling of data gathered by all language versions [14]. The linguistic validation included concept definition, back and forward translations, and cognitive interviews with 55 caregivers of patients with schizophrenia, five per language. Few respondents raised specific cultural concerns or difficulties leading to modifications in the translated version of the SCQ.

The pilot version of the SCQ was included in the PATTERN study, an observational study conducted to describe the course of illness for patients with persistent symptoms of schizophrenia [15]. The present analysis aimed to define the scoring algorithm for the newly developed SCQ that could help to better understand the diverse aspects of caregiver’s experience, and to investigate its measurement properties to ensure that it is a valid and appropriate instrument for use in future studies to assess the impact of care in schizophrenia from the perspectives of caregivers.

## Methods

### Study design

The PATTERN study (ClinicalTrials.gov Identifier: NCT01634542) is an international, multicenter, non-interventional, prospective study of stabilized patients with persistent symptoms of schizophrenia receiving outpatient care [15]. The primary objective was to describe the course of illness for patients with persistent symptoms of schizophrenia. The assessment of the emotional and economic impact and health-related quality of life (HRQL) in caregivers was a secondary objective. Patients included in the study were ≥18 years of age and diagnosed with schizophrenia according to DSM-IV-TR or International Classification of Diseases, 10th revision, documented with the Mini International Neuropsychiatric Inventory, of ≥ 12 months duration before the baseline observation. Caregivers included in the study were a family member/relative or significant other/close friend ≥18 years of age spending more than four hours a week with the patient. Caregivers accompanying a patient to the baseline visit were invited to participate in the study. Once consented, caregivers were invited to complete the SCQ and caregiver's global impression scales (CaGI).

### Assessments

The present analyses were performed using interim data of the PATTERN study. The interim data included 6 months of follow-up data. The caregiver data included the SCQ and CaGI completed at baseline and at 3- and 6-months, and the SF-36 completed only at baseline. The pilot version of the SCQ consisted of 32 items to assess the impact of care experienced by the caregivers of patients with schizophrenia with higher scores representing higher impact. The hypothesized initial structure measured 12 domains: ‘Caregiver roles and responsibilities’; ‘Caregiver perception of patient dependence’; ‘Caregiver perception of level of care they provide’; ‘Exhaustion with caregiving role’; ‘Lack of support’; ‘Impact on caregiver’s daily lives’; ‘Finances’; ‘Impact on social and financial life’; ‘Emotional’; ‘Physical’; ‘Concerns for future’ and ‘Present concerns’. The CaGI severity (CaGI-S) is a scale completed by the caregiver to assess the severity of the symptoms of schizophrenia experienced by the patient: “Please rate the severity of his/her symptoms during the past 4 weeks”. It is rated on a 6-point scale from 1 (no symptoms) to 6 (very severe symptoms). The CaGI improvement (CaGI-I) is a scale for the caregiver to rate the change in severity of symptoms of schizophrenia from baseline: “Overall, how have his/her symptoms changed (if at all) since the beginning of the study (before starting treatment)?” It is rated on a 7-point scale from 1 (very much improved) to 7 (very much worse). The Medical Outcome Survey Short-Form 36 items (SF-36) is a frequently used generic scale to assess aspects of functioning and physical and mental health [16].

Other data included the Positive and Negative Syndrome Scale (PANSS), the Personal and Social Performance (PSP) and the clinician global impression scales (CGI) at baseline and the CGI at 3- and 6-months.

The PANSS is a 30 item, clinician completed scale which assesses the severity of patient’s symptoms and is informed by a clinical interview with patients and their caregivers. In addition to the PANSS total or subdomain scores, 5 factor scores can be calculated: negative symptoms, positive symptoms, disorganized thought, hostility/excitement and anxiety/depression [17]. The PSP is a single-item clinician-rated 100-point scale enabling the determination of small changes in levels of functioning [18]. CGI-Severity (CGI-S) and CGI Improvement (CGI-I) are 7-point clinician-rated scales to measure the overall illness severity from 1 (normal) to 7 (among the most severely ill) and change in symptom severity since baseline scored from 1 (very much improved) to 7 (very much worse) [19].

### Analysis for the validation of the SCQ

The structure of the questionnaire was determined using item response distributions and iterative confirmatory factor analysis (CFA). Fit of the CFA models was evaluated with commonly used goodness-of-fit indices: Root Mean Square Error of Approximation (RMSEA); Standardized Root Mean Residuals (SRMR); Goodness of Fit Index (GFI); Adjusted Goodness of Fit Index (AGFI); Normed Fit Index (NFI); Comparative Fit Index (CFI). The most stringent criteria for goodness-of-fit found in the literature (RMSEA < 0.05; SRMR < 0.05; GFI > 0.90; AGFI > 0.90; NFI > 0.94; CFI > 0.95) were considered [20]; however, these criteria have been reported to potentially be too conservative [21]. CFA model fit was evaluated when a model met some, but not all of these aforementioned criteria demonstrating a consistently solid picture in terms of fit. Iterative CFAs were applied on the item correlation matrix obtained using SCQ data collected at baseline. The first CFA was conducted using the original conceptual framework composed of the SCQ’s 32 items grouped into 12 domains. Then, a series of modifications were made to the model to improve its quality in an iterative approach. Items with low factor loadings were either re-allocated to other domains or deleted from the model. Then it was explored whether the SCQ items could fit a Rasch model, to generate further information that may be used to support decisions on the scoring of the SCQ. A Rating Scale Model was tested for the Humanistic impact supra-domain of the SCQ only, for which a clear underlying unidimensional latent construct could be hypothesized both theoretically and from the empirical results of the CFA.

Construct validity (item concurrent and divergent validity, concurrent and known-group validity), test-retest and internal consistency reliability, and ability to detect change were assessed. Concurrent validity was assessed by Pearson correlation coefficients between SCQ scores and SF-36, PANSS and PSP scores. Clinical validity was assessed by comparing SCQ scores at baseline according to patients’ disease severity assessed using CGI-S, and CaGI-S. Internal consistency reliability of multi-item scores was assessed by Cronbach’s alpha. Test-retest reliability was assessed by interclass correlation coefficient (ICC) between the completion of the SCQ at baseline and month 3 in caregivers who rated the patient’s schizophrenia severity as unchanged. Ability of the SCQ to detect change was assessed by comparing SCQ mean changes in scores from baseline to month 6 according to groups defined by responses to the CaGI-I (Improved group: 'very much improved', 'much improved' and ‘minimally improved’; Stable group: 'no change’; Worsened group: 'minimally worse', 'much worse' and 'very much worse') [22, 23]. Student *t*-tests or one-way ANOVAs were used for the statistical comparisons.

Interpretation guides for the SCQ scores were estimated using both distribution-based and anchor-based methods.

All analyses were performed using SAS statistical software version 9.2 (SAS Institute, Cary, NC, USA), except Rasch model for which RUMM 2030 (RUMM Laboratory, Perth, Australia) was used.

## Results

### Populations

A total of 385 caregivers were recruited in the PATTERN study and among them, 358 had SCQ data. Mean age of patients who were cared for by the included caregivers was 40.0 years. The majority of patients were male (67.6 %). Most patients were rated as moderately (44.9 %) or markedly ill (28.4 %) by their clinicians. From the caregivers’ perspective, 33.5 % of patients had moderate symptoms and 20.9 % had mild symptoms. Detailed demographic and clinical characteristics of the patients are provided in Table 1.

**Table 1:** Description of baseline patient characteristics corresponding to the caregiver analysis set (*N* = 358)

Characteristics		Patients (*n* = 358)
Age (years), Mean (SD)		40.0 (11.6)
Gender, *n* (%)	Male	242 (67.6)
Country, *n* (%)	Argentina	33 (9.2)
	Brazil	22 (6.3)
	Canada	18 (5.2)
	Germany	42 (11.7)
	Spain	92 (26.3)
	France	14 (3.9)
	United Kingdom	29 (8.1)
	Italy	107 (29.9)
Smoking status, *n* (%)	Current smoker	152 (42.5)
	Never smoked	162 (45.2)
	Past smoker	43 (12.0)
PSP, Mean (SD)	Global score	49.8 (19.2)
PANSS, Mean (SD)	Negative symptoms	24.7 (7.3)
	Positive symptoms	23.3 (8.0)
	Disorganized thought	19.4 (6.1)
	Uncontrolled hostility/Excitement	8.2 (3.9)
	Anxiety/Depression	10.5 (3.9)
	Total	86.1 (23.4)
CGI-S, *n* (%)	Normal	6 (1.7)
	Minimally ill	19 (5.3)
	Mildly ill	57 (16.0)
	Moderately ill	160 (44.9)
	Markedly ill	101 (28.4)
	Severely ill	12 (3.4)
	Among the most severely ill	1 (0.3)
CaGI-S, *n* (%)	No symptoms	50 (14.1)
	Very mild symptoms	68 (19.2)
	Mild symptoms	74 (20.9)
	Moderate symptoms	119 (33.5)
	Severe symptoms	40 (11.3)
	Very severe symptoms	4 (1.1)

*PSP* Personal and Social Performance Scale, *PANSS* Positive and Negative Syndrome Scale, *CGI-S* Clinician Global Impression – Severity, *CGI-S* Caregiver Global Impression – Severity

### Definition of scoring algorithm of the SCQ

Since responses to SCQ items were compulsory in the electronic data collection system, there were no missing data. A floor effect was observed for almost all items; six items had over 50 % of caregivers giving the lowest value on the response scale. As the initial model tested in the CFA, which was based on the qualitative research and included 12 domains, did not demonstrate a fully acceptable fit (RMSEA: 0.08; SRMR: 0.07; GFI: 0.79; AGFI: 0.76; NFI: 0.72; CFI: 0.87) alternative models were examined.

**Fig. 1:**
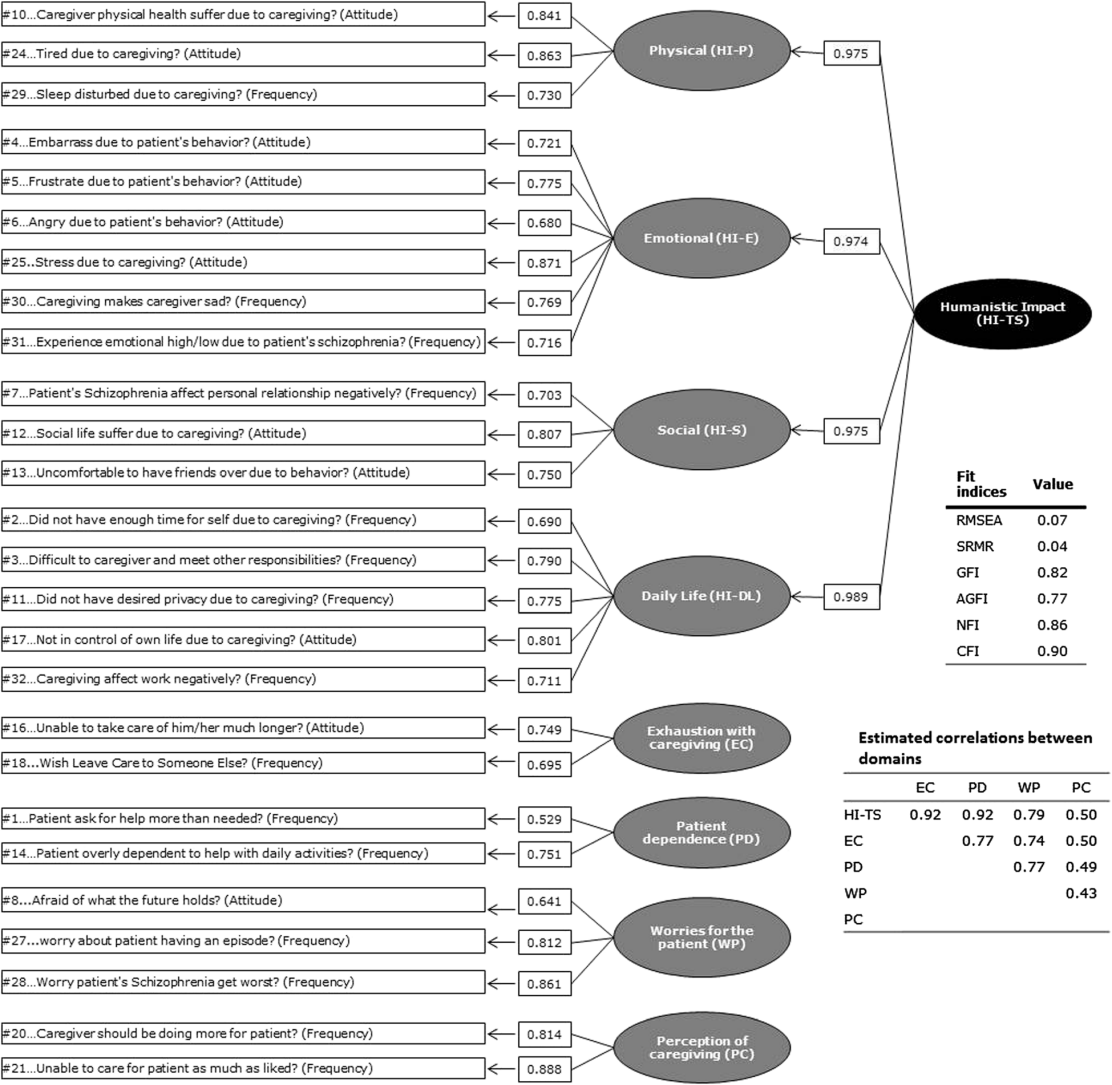
CFA results of the final model of the SCQ linking items, concepts, and the total “Humanistic impact” at baseline in the caregiver analysis set (*N* = 358); RMSEA: Root Mean Square Error of Approximation; SRMR: Standard Root Mean square Residuals; GFI: Goodness of Fit Index; AGFI: Adjusted Goodness of Fit Index; NFI: Normed Fixed Index; CFI: Comparative Fit Index

The results of the final CFA model of the SCQ are displayed in Fig. 1. Even though none of the goodness-of-fit indices met the stringent criteria, considering the overall picture of the fit indices, this final model was deemed overall to be acceptable. Item 19 (“How often did you feel uncertain about how to care for him/her?”)' and item 26 (“How difficult was it to get him/her to take his/her medication?”) were not retained in the SCQ as they did not fit well within their domains with low factor loadings and were deemed to capture concepts central enough to warrant single-item scores. Items 1, 2, 6, 14 and 18 were retained in the questionnaire based on qualitative assessment of their content, despite their imperfect fit to the model. Items 9 and 15 pertaining to financial aspects were separated into two different single item scores as they covered two distinct concepts.

A two component structure was sketched for the SCQ. The first component, named ‘Humanistic impact’ supra-domain, assesses the direct impact on the individual caregiver and includes four subdomains (‘Humanistic impact - Social’, ‘Humanistic impact - Emotional’, ‘Humanistic impact - Daily Life’, and ‘Humanistic impact - Physical’) plus an overall assessment (‘Humanistic Impact - Total score’). The second part of the questionnaire assesses all other aspects of the caregiver experience which does not directly reflect its personal impact, including eight domains: ‘Overall difficulty of caregiving’; ‘Patient dependence’; ‘Perception of caregiving’; ‘Exhaustion with caregiving’; ‘Feeling alone’; ‘Financial dependence of the patient’; ‘Financial impact of caregiving’; ‘Worries for the patient’.

**Fig. 2:**
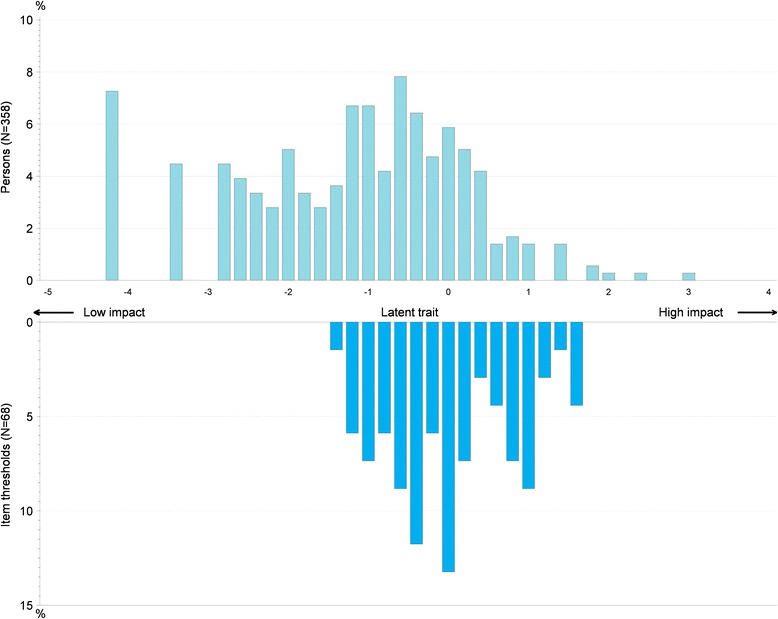
Person-item distribution of SCQ items of the Humanistic impact from the Rating Scale Model with items scored with 5 categories at baseline (*N* = 358)

A Rasch model was applied to the Humanistic Impact supra-domain only. The 11-point rating scale led to several disordered thresholds but recoding the responses in fewer categories led to a model without any disordered thresholds. Items 24 and 25 had residuals greater than 2.5 in absolute value, together with a non-significant chi-square test, indicating poor fit to the model. The Person-Item map resulting from this analysis is presented in Fig. 2. SCQ items cover part of the latent trait (i.e., Humanistic impact) on which most individuals are located. The map also shows that the locations corresponding to very high Humanistic impact (at the extreme right end of the x-axis) and mild impact (at the extreme left end of the x-axis) are not adequately covered by SCQ items. While the former corresponds to only a handful of individuals, there were about one third of caregivers for whom the Humanistic impact was absent or too mild to be accurately captured by the SCQ items.

Based on the observation of response distribution and results of the Rasch model, responses were recoded to a 0 to 4 scale as follows: 0-1 responses were recoded to 0; 2-3-4 to 1; 5-6 to 2; 7-8 to 3; 9-10 to 4. Scores of the SCQ were obtained by a sum of the recoded item scores, linearly transformed to a 0 to 100 range. Higher scores indicated greater impact.

### Assessment of measurement properties of the SCQ

#### Reliability

SCQ domain scores are described at baseline in Table 2, together with results for reliability assessment. For almost all SCQ domain scores, Cronbach’s alpha and ICC demonstrated very good reliability.

**Table 2:** Description of SCQ scores, reliability, item convergent and discriminant validity, and meaningfulness

			Reliability		Confirmation of the structure			Meaningfulness		
								Distribution-based methods	Anchor-based methods	
									Patient minimally improved	Patient minimally worsened
SCQ domains	# of item	Mean Score (SD)	Cronbach’s alfa*	ICC*	Item-domain correlations^a^	# items meeting convergent validity^b^	# items meeting discriminant validity^c^	SD $\times \sqrt{\left(1-\alpha \right)}$	Mean change	Mean change
HI-TS	17	27.8 (23.4)	0.96	0.87	-	-	-	5.13	-3.96	7.67
HI-P	3	27.0 (27.3)	0.85	0.82	0.62-0.75	100 %	33 %	11.09	-6.75	10.42
HI-E	6	30.0 (24.8)	0.89	0.81	0.63-0.75	100 %	83 %	8.59	-2.23	8.78
HI-S	3	26.4 (26.1)	0.80	0.75	0.60-0.66	100 %	0 %	12.21	-3.16	1.49
HI-DL	5	26.5 (24.3)	0.87	0.81	0.59-0.76	100 %	40 %	9.26	-4.83	8.39
EC	2	19.9 (24.7)	0.69	0.76	0.49	100 %	100 %	14.37	-0.86	3.13
FA	1	32.0 (36.0)	NA	0.63	NA	NA	NA	NA	-6.90	8.04
PD	2	36.7 (27.5)	0.57	0.75	0.37	0 %	0 %	18.64	-8.62	6.25
WP	2	42.0 (28.7)	0.70	0.75	0.54-0.71	100 %	100 %	12.93	-7.04	6.55
PC	3	34.0 (29.9)	0.79	0.71	0.71	100 %	100 %	12.45	-1.29	4.02
FDP	1	37.9 (39.6)	NA	0.70	NA	NA	NA	NA	-8.62	-2.68
FIC	1	26.0 (32.1)	NA	0.75	NA	NA	NA	NA	0.86	1.79
ODC	1	28.6 (31.6)	NA	0.75	NA	NA	NA	NA	-6.90	16.96

*SD* Standard deviation; α = reliability coefficient (Cronbach’s α); *ICC* Intraclass correlation coefficient, *NA* not applicable as single-item dimension, *HI-TS* Humanistic impact – total score, *HI-P* Humanistic impact - Physical, *HI-E* Humanistic impact - Emotional, *HI-S* Humanistic impact - Social, *HI-DL* Humanistic impact - Daily life, *EC* Exhaustion with caregiving, *FA* Feeling alone, *PD* Patient Dependence, *WP* Worries for the patient, *PC* Perception of caregiving, *FDP* Financial dependence of the patient, *FIC* Financial impact of caregiving, *ODC* Overall difficulty of caregiving

Description of SCQ scores, internal consistency reliability and item convergent and discriminant validity data were obtained at baseline in the caregiver analysis set (*N* = 358). Test-retest reliability data were obtained between baseline and month 3 in caregivers whose patient’s severity was stable between the two assessments (*N* = 147). Analyses supporting meaningful change in SCQ domain scores were calculated using distribution-based methods at baseline in the caregiver analysis set (*N* = 358) and anchor-based methods in patients minimally improved at Month 6 (*N* = 58) and patients minimally worsened (*N* = 28)

*Reliability coefficient is considered acceptable if > 0.70

^a^Range of correlations between each item and its domain score

^b^Item convergent validity criterion: correlation between an item and its domain score should be ≥0.40

^c^Item discriminant validity criterion: each item should correlate higher with its domain score than with the other domain scores

#### Construct validity

The SCQ domain structure was supported by good item convergent and discriminant validity (Table 2). All items met the item convergent validity criterion (i.e., were well correlated with their own dimension), except for the ‘Patient dependence’ domain (0.37). The four scores exploring different aspects of humanistic impact had imperfect item discriminant validity, probably due to the strong association between these domains, which reflects the existence of a single underlying construct, Humanistic impact.

Low to moderate correlations (0.10 to 0.60) between SCQ domain scores and SF-36 domain scores were observed (Table 3). Scores pertaining to humanistic impact showed moderate correlations with ‘Vitality’, ‘Social Functioning’, ‘Mental Health’ domain scores and Mental Component Score (MCS), and low correlation with the other SF-36 scores related to Physical Functioning or Pain. The score ‘Worries for the patient’ also had moderate correlation with the scores assessing mental health (‘Mental health’ and MCS).

**Table 3:** Concurrent validity - Pearson correlation coefficients between the SCQ domains and the concurrent questionnaires (SF-36, PANSS and PSP) at baseline in the caregiver analysis set (*N* = 358)

		SCQ domains												
Scales	Domains	HI-TS	HI-P	HI-E	HI-S	HI-DL	ODC	PD	PC	EC	FA	FDP	FIC	WP
SF-36	Physical Functioning	-0.22	-0.21	-0.19	-0.24	-0.20	-0.15	-0.20	-0.07	-0.15	-0.11	-0.11	-0.23	-0.21
	Role Physical	-0.20	-0.20	-0.15	-0.22	-0.20	-0.16	-0.15	-0.11	-0.17	-0.08	-0.06	-0.20	-0.18
	Bodily Pain	-0.24	-0.23	-0.21	-0.22	-0.22	-0.16	-0.17	-0.14	-0.12	-0.10	-0.10	-0.22	-0.27
	General Health Perceptions	-0.39	-0.39	-0.34	-0.37	-0.36	-0.27	-0.18	-0.20	-0.29	-0.23	-0.12	-0.25	-0.34
	Vitality	**-0.46**	**-0.45**	**-0.40**	**-0.43**	**-0.45**	-0.34	-0.24	-0.23	-0.31	-0.29	-0.15	-0.25	-0.38
	Social Functioning	**-0.43**	**-0.43**	-0.37	**-0.41**	**-0.41**	-0.33	-0.23	-0.29	-0.33	-0.25	-0.19	-0.32	-0.37
	Role Emotional	**-0.41**	-0.38	-0.38	-0.37	-0.39	-0.28	-0.20	-0.26	-0.29	-0.21	-0.14	-0.28	-0.31
	Mental Health	**-0.56**	**-0.51**	**-0.53**	**-0.51**	**-0.51**	**-0.42**	-0.32	-0.26	-0.37	-0.32	-0.20	-0.37	**-0.51**
	PCS	-0.15	-0.16	-0.11	-0.17	-0.14	-0.10	-0.13	-0.07	-0.11	-0.06	-0.06	-0.17	-0.17
	MCS	**-0.56**	**-0.53**	**-0.52**	**-0.50**	**-0.53**	**-0.42**	-0.27	-0.32	-0.39	-0.33	-0.20	-0.34	**-0.46**
PANSS	Positive symptoms	0.31	0.26	0.31	0.27	0.30	0.27	0.29	0.17	0.28	0.25	0.18	0.24	0.24
	Negative symptoms	0.22	0.19	0.19	0.19	0.25	0.21	0.24	0.07	0.24	0.11	0.16	0.24	0.19
PSP	PSP total	-0.21	-0.18	-0.16	-0.22	-0.24	-0.23	-0.21	-0.11	-0.16	-0.10	-0.17	-0.17	-0.11

*PSP* Personal and Social Performance Scale, *PANSS* Positive and Negative Syndrome Scale, *HI-TS* Humanistic impact – total score, *HI-P* Humanistic impact - Physical, *HI-E* Humanistic impact - Emotional, *HI-S* Humanistic impact - Social, *HI-DL* Humanistic impact - Daily life, *ODC* Overall difficulty of caregiving, *PD* Patient Dependence, *PC* Perception of caregiving, *EC* Exhaustion with caregiving, *FA* Feeling alone, *FDP* Financial dependence of the patient, *FIC* Financial impact of caregiving, *WP* Worries for the patient

Pearson's correlations; In bold, 0.40 < correlations < 0.60

Low correlations were observed between SCQ domain scores and the PANSS and PSP domain scores (-0.24 to 0.31) (Table 3).

**Fig. 3:**
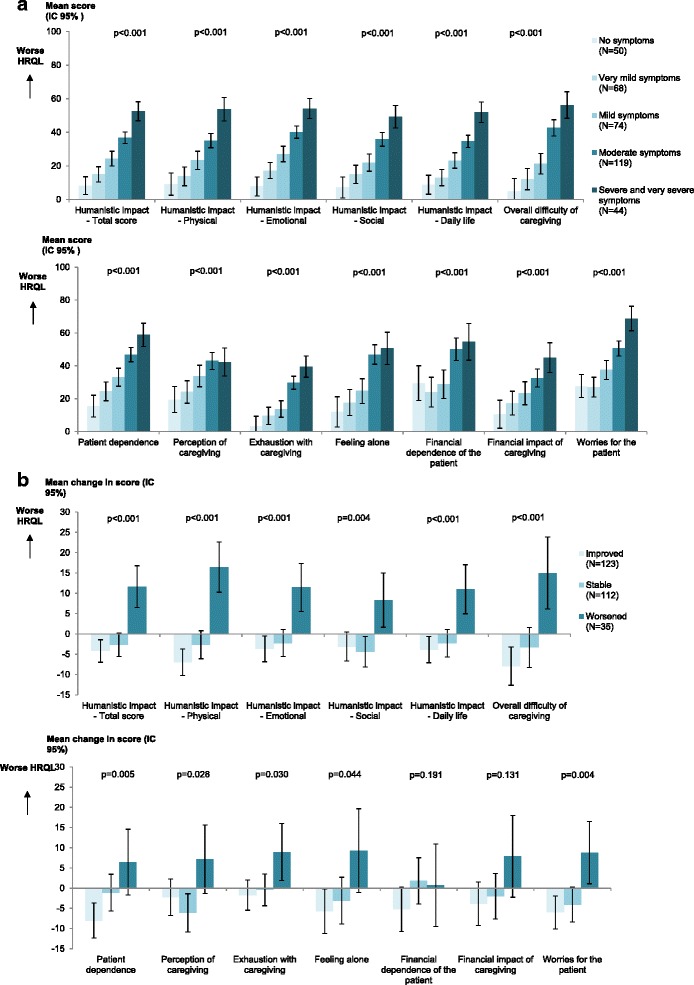
Measurement properties of SCQ: **a** Clinical validity - comparison of the SCQ domain scores according to CaGI-S at baseline in the caregiver analysis set (*N* = 358); **b** Ability to detect change - comparison of the change in SCQ domain scores according to CaGI-I between baseline and 6 months in patients with SCQ data at baseline and Month 6 (*N* = 270)

The severity of schizophrenia perceived by the caregiver (CaGI-S) was associated with all SCQ domain scores (*p* < 0.001), with higher mean scores when the disease was rated as more severe (Fig. 3). Similar findings were observed when comparing SCQ scores to the level of severity of schizophrenia assessed using the CGI-S (data not shown).

#### Ability to detect change

For all domains except ‘Financial dependence of the patient’, SCQ scores decreased for caregivers of patients whose schizophrenia improved and increased for caregivers of patients whose schizophrenia worsened. (Fig. 3) The increase was particularly visible in the scores pertaining to Humanistic impact. Overall, this pattern of results supports the ability of the SCQ to detect changes in the caregiver experience.

### Interpretation of SCQ scores

Meaningful change for SCQ domain scores estimated using both distribution-based and anchor-based methods are presented in Table 2. The distribution-based values ranged from 5.1 to 19.8. Anchor-based values, obtained using the severity of the patient’s schizophrenia assessed using the CaGI as an anchor, ranged between 0.86 and -8.62 for improvement and -2.68 and 16.96 for worsening. However, these anchor-based values should be interpreted cautiously because, for some domains such as financial aspects or perception of care provided, the anchor used (patient schizophrenia severity) is likely not well correlated with the actual impact on the caregiver, making the resulting value not appropriate.

## Discussion

The objective of this study was to create a scoring algorithm and assess the measurement properties of the SCQ, a newly developed instrument to assess the impact of caregiving for a person with schizophrenia from the perspectives of caregivers. The SCQ scoring algorithm was developed using the a priori conceptual model of the SCQ from the qualitative research phase [13] and empirical results from the observational PATTERN study. Analyses revealed two distinct constructs assessed by the SCQ: ‘Humanistic impact,’ comprising the social, emotional, daily life and physical impact on the individual caregiver; and aspects related to the caregiving role, such as perceptions of the caregiver towards caregiving (e.g. feeling alone, dependence of the patient, or exhaustion with caregiving) or financial aspects.

The measurement properties (reliability, validity and ability to detect change) demonstrated by the SCQ in the PATTERN study were excellent. The SCQ showed good reliability, with most reliability coefficients above the recommended threshold of 0.7 [24]. SCQ construct validity was consistently supported. Firstly, item convergent and divergent validity was fully satisfactory; only the subdomain scores within the Humanistic impact supra-domain showed imperfect item divergent validity. This may have been due to their relationship with the common underlying concept Humanistic impact. However, in order to document salient aspects of the impact of caregiving on caregivers these subdomains were retained. Secondly, patients with more severe symptoms of schizophrenia according to the clinicians’ overall severity rating were consistently associated with higher SCQ scores indicating more impact for the caregiver. Thirdly, an expected pattern of association was found between SCQ scores and other measures of severity of patient’s schizophrenia and caregiver HRQL. In particular, the salience of the psychological and social aspects of caregiving were marked by the the highest observed correlations between the measures of the Humanistic impact (including subdomain scores) and the SF-36 score pertaining to the mental and social aspects. In addition, the correlations between these SCQ scores and the SF-36 ‘Vitality’ score may reflect the critical role of tiredness on the impact on caregivers of patients with schizophrenia [7]. Finally, most SCQ scores demonstrated clear changes as the patient’s schizophrenia symptoms increased in severity and, a more subtle change, when symptoms of schizophrenia improved. These results demonstrate that changes in the patient’s symptoms of schizophrenia could also translate into changes on the impact on their caregiver. Based on these findings, an interesting follow-up analysis would be to further explore the association between the change in severity of schizophrenia symptoms and impact on the caregivers, by studying the association between the SCQ scores and changes in measures of schizophrenia symptoms such as the PANSS.

PATTERN data also offered an opportunity to generate preliminary information to guide interpretation of the SCQ scores. Changes of between 9 and 13 points for the subdomains of the humanistic impact supra-domain and slightly lower (about 6 or 7) for the Humanistic impact - Total score appeared to be meaningful. Interestingly, the anchor-based method suggested different values for improvement and worsening. Importantly, these results should be interpreted with caution as 1) they were obtained either using a distribution-based approach or using the severity of patient schizophrenia symptoms (and not a true assessment of the change in the caregiver experience) as an anchor, and 2) were obtained with a fairly small number of caregivers. Further results will be needed to consolidate the guidance for the interpretation of SCQ scores.

Some instruments were already available to assess the impact on caregivers of patients with schizophrenia: the Schizophrenia Caregiver Quality of Life questionnaire (SCGQoL) [25], the Experience of Caregiving Inventory (ECI) [26] and the Involvement Evaluation Questionnaire (IEQ) [27]. However, the SCQ has several advantages in comparison as it builds on the ZBI, which is a widely-used standard assessment of caregiver impact, and followed questionnaire development best-practices beginning with qualitative research involving a substantial number of caregivers of patients with schizophrenia [13]. In addition, the SCQ demonstrated strong, reliable measurement properties possibly as a result of psychometrically sound questionnaire development. While the SCQ may focus on the level of negative impacts a caregiver can experience, a specific instrument as for example the Scale for Positive Aspects of Caregiving Experience (SPACE) [28] could be used to assess positive aspects of care in schizophrenia and thus provide a comprehensive picture of caregiver experience. Finally, the SCQ is also available in a number of language versions, which have been obtained through a high standard of linguistic validation [14], and could be used to facilitate research in multinational settings.

Some remarkable findings on the SCQ may deserve further attention in the future. First, the SCQ may not be able to accurately measure when only a very mild or no impact is experienced by some caregivers. This was shown by Rasch modeling and consistent floor effects observed for all items of the questionnaire. This may present an issue if the questionnaire is administered to caregivers experiencing only mild impact of caregiving. The SCQ may also under-identify improvement in the impact on the caregiver when the baseline impact is already minor. Further research may be needed to identify the markers of the mild impact of caregiving, which could be useful in developing items allowing better assessment for caregivers with mild caregiving impact. Secondly, the SCQ response scale was modified compared to the ZBI from a 5-point response scale to an 11-point Numeric Rating Scale (NRS). Our analyses led to the definition of SCQ scores based on recoding of the 11-point response scales into 5 categories. As the SCQ includes both items measuring frequency, which are probably most naturally assessed with a 5-point response scale, and items measuring intensity, for which an 11-point NRS may be more appropriate, using an 11-point NRS and recoding the responses at the scoring stage is still a valid option in order to use a consistent response scale throughout the instrument.

The PATTERN study was an observational study primarily designed to assess the course of symptoms of schizophrenia, with the caregiver component of the study as an ancillary study. Due to this, the information on caregivers who completed the questionnaire was only partial, making the characterization of the analysis sample less accurate than generally expected. This also prevented the analysis of Differential Item Functioning (DIF) for the SCQ items in terms of gender, age or relation with the patient (i.e., parent, spouse or sibling) of the caregiver. In addition, no natural “anchor” was collected to characterize the change in the caregiver experience, resulting in a less robust detection in change in caregiving impacts, since it relied only on the change in the patient’s overall health status. Finally, the electronic data collection method ensured the completion of the SCQ items and therefore any information on the understanding and relevance of the items potentially showed by non-response could not be captured. All these questions would be worth exploring in future research.

## Conclusions

The SCQ was shown to be an instrument allowing the comprehensive assessment of the experience of caregivers of patients with schizophrenia through 30 items that allow the calculation of 9 multi-item scores and 4 single item scores that all demonstrated very good measurement properties. This new algorithm should offer an approach to describe the diverse aspects of caregiver’s experience. The care for patients with schizophrenia relies more and more heavily on informal caregivers. Hence, the assessment of the impact of schizophrenia, and of any intervention linked to management of patients should be holistic. Such a holistic approach would only be complete with perspectives from the patient’s social ecology, which necessarily involves the caregivers. The SCQ, as a schizophrenia specific measure of caregiver impact, will be an instrument of choice in this context and should therefore be extremely useful in future schizophrenia studies.

## Abbreviations

AGFI, adjusted goodness of fit index; CaGI-I, caregiver's global impression of improvement; CaGI-S, caregiver's global impression of severity; CFA, confirmatory factor analysis; CFI, comparative fit index; CGI-I, clinician global impression of improvement; CGI-S, clinician global impression of severity; ECI, experience of caregiving inventory; GFI, goodness of fit index; DIF, differential item functioning; HRQL, health-related quality of life; ICC, interclass correlation coefficient; IEQ, involvement evaluation questionnaire; MCS, mental component score; NFI, normed fit index; NRS, numeric rating scale; PANSS, positive and negative syndrome scale; PSP, personal and social performance; RMSEA, root mean square error of approximation; SF-36, medical outcome survey short-form 36 items; SCQ, Schizophrenia Caregiver Questionnaire; SGQoL, schizophrenia caregiver quality of life questionnaire; SRMR, standardized root mean residuals; ZBI, Zarit burden interview
